# Supplementary material for: Identification of photoperiod-induced specific miRNAs in the adrenal glands of Sunite sheep (Ovis aries)
Source: Front Vet Sci. 2022 Jul 22;9:888207. doi: 10.3389/fvets.2022.888207 (PMC9354845; doi:10.3389/fvets.2022.888207)
Supplement: Supplementary file 1 [file Data_Sheet_1.ZIP › Supplementary file/miRDeep2 file.zip!/miRDeep2 file/Know_Structure/LP42A3/oar-mir-329b.pdf]

miRBase precursor : oar-mir-329b
Total read count : 3779
oar-miR-329b-5p read count : 42
oar-miR-329b-3p read count : 3722
remaining reads : 15

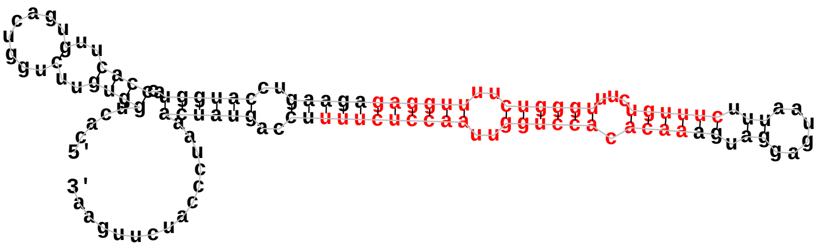

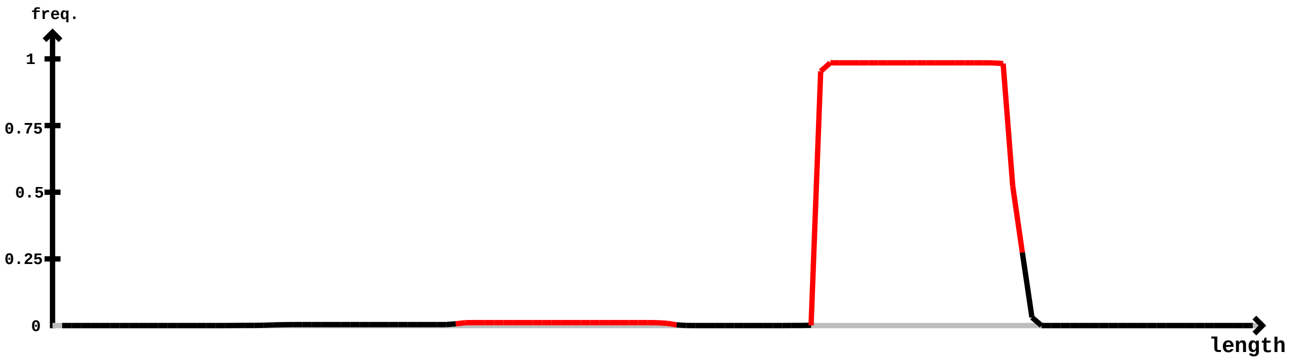


```
                                                                     oar-miR-329b-3p
                                         oar-miR-329b-5p
5'- cacugguguucuggucaguguucacccaugguaccugaagagagguuuucuggguuucuguuucuuuaaugaggaugaaacacaccugguuaaccucuuuuccaguaucaaaucccaucuugaa   -3'  exp
    ....((((..(........)..))))..((((((..((((((((((..((((((...((((((.((......)).)))))).))))))..))))))))))..))))))................   reads  mm
    .................guguucacccaugguaccugaaga...................................................................................   1      0
    ..................uguucacccaugguaccugaaga...................................................................................   1      0
    ....................uucacccaugguaccugaaga...................................................................................   2      0
    .....................ucacccaugguaccugaaga...................................................................................   5      0
    ......................cacccaugguaccugaaga...................................................................................   3      0
    .......................acccaugguaccugaaga...................................................................................   2      0
    ........................................agagguuuucuggguuucuguu..............................................................   5      0
    ........................................agagguuuucuggguuucuguuu.............................................................   6      0
    .........................................gagguuuucuggguuucugu...............................................................   2      0
    .........................................gagguuuucuggguuucuguu..............................................................   3      0
    .........................................gagguuuucuggguuucuguuu.............................................................   15     0
    .........................................gagguuuucuggguuucuguuuc............................................................   9      0
    .........................................gagguuuucuggguuucuguuucu...........................................................   1      0
    ..........................................agguuuucuggguuucuguuu.............................................................   1      0
    ...........................................................................ugaaacacaccugguuaaccucu..........................   1      0
    .............................................................................aaacacaccugguuaaccucu..........................   1      0
    .............................................................................aaacacaccugguuaaccucuu.........................   2      0
    ..............................................................................aacacaccugguuaaccu............................   1      0
    ..............................................................................aacacaccugguuaaccuc...........................   9      0
    ..............................................................................aacacaccugguuaaccucu..........................   1724   0
    ..............................................................................aacacaccugguuaaccucuu.........................   939    0
    ..............................................................................aacacaccugguuaaccucuuu........................   877    0
    ..............................................................................aacacaccugguuaaccucuuuu.......................   49     0
    ...............................................................................acacaccugguuaaccucu..........................   10     0
    ...............................................................................acacaccugguuaaccucuu.........................   1      0
    ...............................................................................acacaccugguuaaccucuuu........................   42     0
    ...............................................................................acacaccugguuaaccucuuuu.......................   67     0
```
